# Mechanical Properties of Austenitic Stainless Steel Made by Additive Manufacturing

**DOI:** 10.6028/jres.119.015

**Published:** 2014-10-10

**Authors:** William E Luecke, John A Slotwinski

**Affiliations:** National Institute of Standards and Technology, Gaithersburg, MD 20899

**Keywords:** additive manufacturing, hardness, Lueders bands, tensile testing, UNS 174100

## Abstract

Using uniaxial tensile and hardness testing, we evaluated the variability and anisotropy of the mechanical properties of an austenitic stainless steel, UNS S17400, manufactured by an additive process, selective laser melting. Like wrought materials, the mechanical properties depend on the orientation introduced by the processing. The recommended stress-relief heat treatment increases the tensile strength, reduces the yield strength, and decreases the extent of the discontinuous yielding. The mechanical properties, assessed by hardness, are very uniform across the build plate, but the stress-relief heat treatment introduced a small non-uniformity that had no correlation to position on the build plate. Analysis of the mechanical property behavior resulted in four conclusions. (1) The within-build and build-to-build tensile properties of the UNS S17400 stainless steel are less repeatable than mature engineering structural alloys, but similar to other structural alloys made by additive manufacturing. (2) The anisotropy of the mechanical properties of the UNS S17400 material of this study is larger than that of mature structural alloys, but is similar to other structural alloys made by additive manufacturing. (3) The tensile mechanical properties of the UNS S17400 material fabricated by selective laser melting are very different from those of wrought, heat-treated 17-4PH stainless steel. (4) The large discontinuous yielding strain in all tests resulted from the formation and propagation of Lüders bands.

## 1. Introduction

Unlike traditional manufacturing processes such as turning and milling that produce parts by removing unwanted material from a larger piece, additive manufacturing (AM) processes build parts one thin layer at a time. This can be done in a variety of ways, such as sintering of powder via laser or electron beams, extrusion and deposition of polymer via a heated orifice, or selective curing of liquid photopolymers. These processes can all produce complex, high-value parts that cannot be fabricated with traditional material removal processes, can accomplish this without tooling, and with the ability to go almost directly from a digital design to part. The full vision of additive manufacturing includes using these processes to produce complex, customized metal parts for use in high-stress, mission-critical aerospace applications, such as jet engine components and turbine blades, where revolutionary, weight-saving part designs that include complex interior structures could revolutionize the manufacturing industry.

Additive manufacturing successes have received significant media attention in the popular press recently, and while additive manufacturing is already producing customized metal parts in niche applications such as dental implants [1] and demonstrating truly impressive capabilities that generate lots of societal excitement for the future of additive manufacturing [2,3], the full benefits of additive manufacturing are not yet realized in a widespread way across the manufacturing industry. Several technical challenges must first be overcome, including a lack of robust, design-allowable materials property data and inconsistent AM processes that result in variable materials properties [4,5].

In this paper we report some baseline mechanical property data for UNS S17400 stainless steel test specimens produced on a commercial laser-sintering machine. Specifically we examined

- the build-to-build repeatability and consistency of the tensile properties;
- the dependence of mechanical properties on the build orientation;
- the effects of post-processing heat treatment on mechanical properties, and
- the variation of mechanical properties across the build plate.

## 2. Experimental Details

### 2.1 Material: UNS S17400 Stainless Steel

The stainless steel test specimens were produced at NIST using selective laser melting (SLM), using an EOSINT M 270 laser-sintering system^1^ using the standard EOS build conditions that deposit layers 20 μm thick in a checkerboard pattern with rotation between layers. The chemical composition of the material conforms to UNS S17400 [6,7], which is the nominal composition for for 17-4PH stainless steel, a corrosion-resistant martensitic stainless steel whose strength is increased by copper precipitation. The machine manufacturer supplied the powder. A separate manuscript summarizes relevant powder parameters [8].

Because the manufacturer recommends heat treating stainless steel parts for 1 h at 650 °C to relieve residual stress, some parts were heat-treated in an electrical resistance heated convection furnace in air. The specimens were contained in a nominally sealed stainless steel bag that was inserted in the cold furnace, which was then brought to temperature in about one hour.

### 2.2 Tensile Testing

#### 2.2.1 Specimen

**Fig 1:**
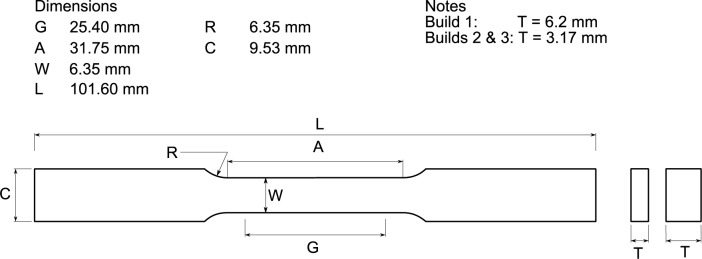
Tensile specimen.

**Fig. 2:**
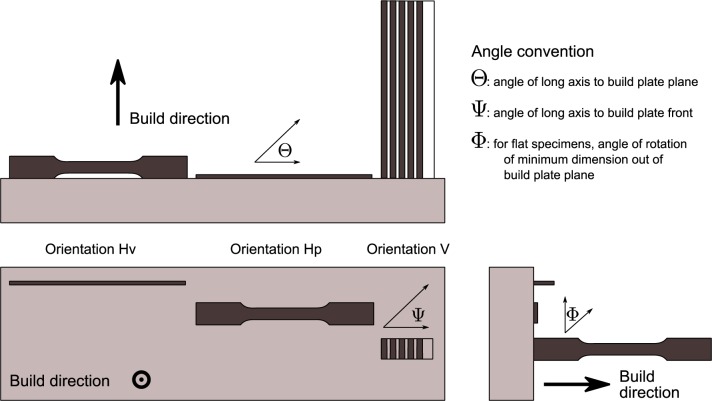
Projection diagram of the three build orientations for the tensile specimens.

All tests used an ASTM E8 [9] subsize sheet specimen, Fig. 1. Three possible specimen orientations exist with respect to the build direction, shown in Fig. 2. Two orientations, Hv and Hp, orient the build direction perpendicular to the long axis of the specimen. In orientation “Hp” (for plan view), the build direction is normal to the plane of the specimen tab. In orientation “Hv” the build direction is normal to the side (narrowest dimension) of the specimen. Finally, in vertically oriented specimens, denoted “V,” the build direction proceeds along the tensile axis of the specimen. In the vertical orientation the individual specimens are sliced from the block with electrical discharge machining (EDM). One face of the Hp orientation specimens is the final layer added, while the opposite face is produced when the specimen is cut from the build block with electrical-discharge machining. Similarly for the Hv orientation, one narrow side of the specimen has a finished face, while the opposite side has the remains of the support structures. Build 1 employed Hv orientation, while builds 2 and 3 employed the Hp orientation. The specimens were fabricated to near net shape. In build 1, it was necessary to remove the support structures from one face of the specimen, which may increase the dimensional uncertainty, and therefore the uncertainty of the reported strengths. For builds 2 and 3 we made no effort to machine or grind the faces after fabrication. Because the fabrication is so different than for wrought materials, no correspondence can be made between the traditional longitudinal, transverse, and short-transverse directions.

#### 2.2.2 Tensile Tests

All tensile tests were conducted in position control in a servo-hydraulic test machine equipped with mechanical wedge grips. The extensometers met ASTM E83 [10] classification B-2. Build 1 employed an extensometer with *G* = 12.45 mm, and builds 2 and 3 employed one with *G* = 25.4 mm. Because the maximum measurable strain without resetting the extensometer was about *e* = 0.2, the extensometer was frequently removed before the specimen reached the tensile strength. Tests followed ASTM E8, but the extension rate was not increased after yield, and the strain rate for the build 2 tests was about ten times higher than in builds 1 and 2.

Table 1 summarizes the distribution of orientations, heat treatments across the three build rounds.

### 2.3 Hardness Testing

Hardness tests employed the Rockwell A scale on an analog Wilson/Rockwell 3JR hardness test machine with a flat anvil. The support structures on the bottom of the test specimens were filed flat before testing Each of 16 parts received three hardness tests; the indents were spaced to 10 mm sided triangle in the center of the part. After every three parts (nine tests), a standard test block (51.2 ± 1 HRA (*k* = 2), NIST traceable) was tested to ensure repeatability. The standard test block repeatability was less than the machines indicator dial resolution, approximately 0.25 HRA. Tests were conducted both before and after heat treating at *T* = 650°C in Ar gas.

## 3. Results

### 3.1 Tensile Stress-Strain Curves

Figures 4, 5, and 6 summarize the tensile stress-strain curves from the three builds in the two orientations and two heat-treatment conditions.

**Fig. 3:**
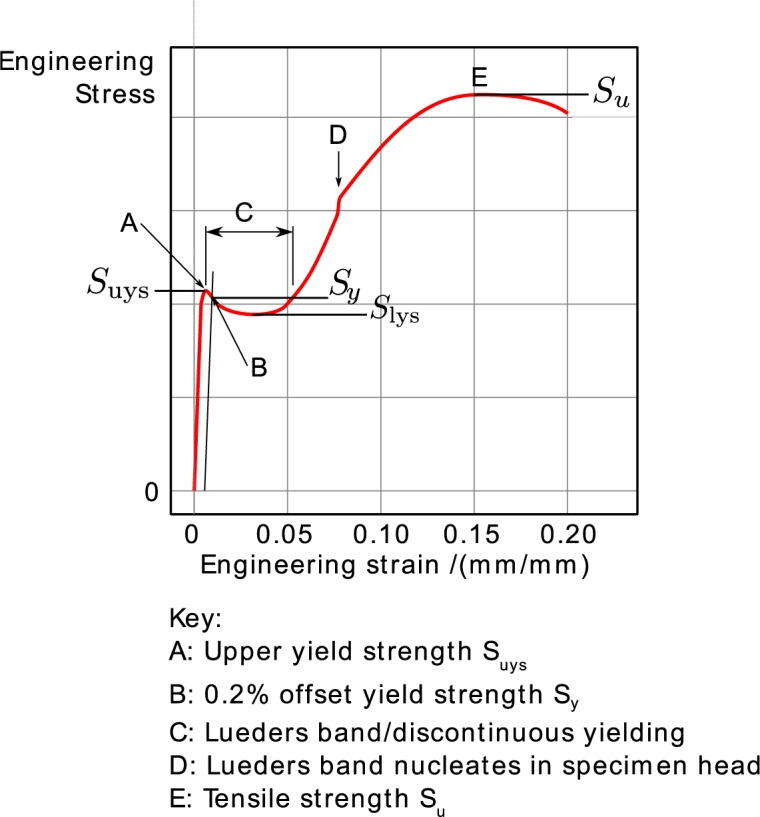
Schematic diagram of the stress-strain curve.

The stress-strain curves of all specimens are similar. Figure 3 is a schematic diagram of the important features of each curve. Each stress-strain curve has a diffuse upper yield strength, followed by a long region of yield point elongation, also called a Lüders strain. In some specimens the structure of the strain increment associated with discontinuous yielding is quite repeatable. In addition, there is often a small discontinuity in the stress-strain curve where we suspect that a second Lüders band nucleates in the specimen tab. Because the specimen tab is only 1.5 times the gauge width, see Fig. 1, strain hardening eventually raises the strength of the gauge section above that of the tab, which forces the tab to yield as well.

**Fig. 4:**
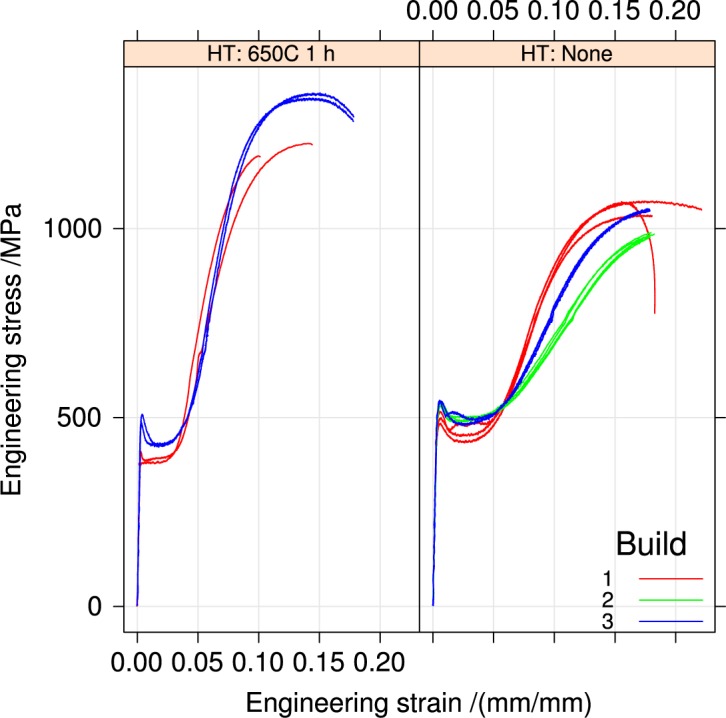
Tensile stress-strain curves for the three builds, horizontal orientation, separated by heat treatment.

Figure 4 compares as-received specimens to specimens heat-treated in air at 650 °C for 1 h for the three builds. Heat treating reduces all the yield strengths, but increases the tensile strength *S_u_*. Builds 2 and 3 are similar, and different from build 1.

**Fig. 5:**
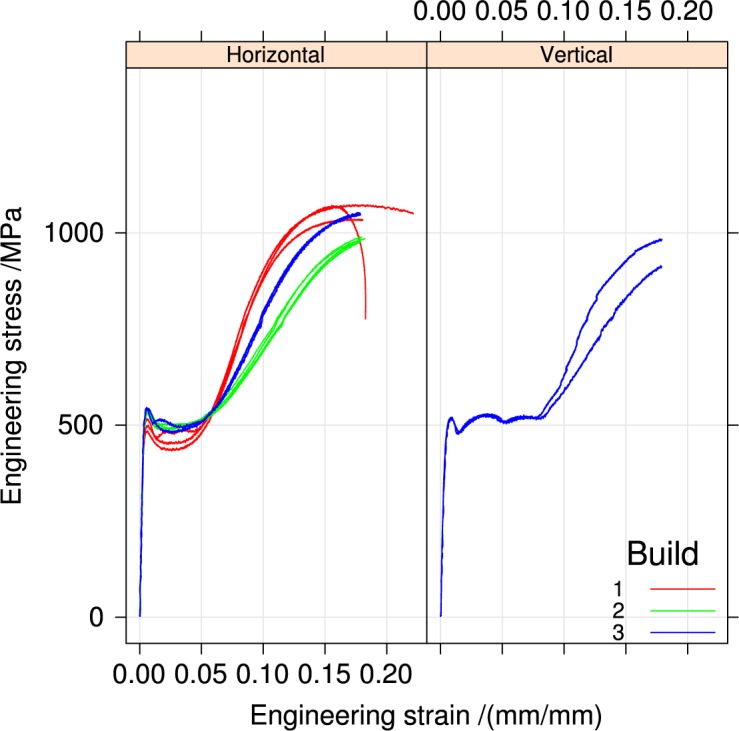
Tensile stress-strain curves for the builds, separated by orientation, for the un-heat-treated condition.

Figure 5 replots the stress-strain data but differentiates it by build orientation for specimens that were not heat treated. Only build 3 contains vertically oriented specimens. The discontinuous yielding in build 2 Vertical specimens is longer, and its structure differs from the horizontal specimens of all three builds.

**Fig. 6:**
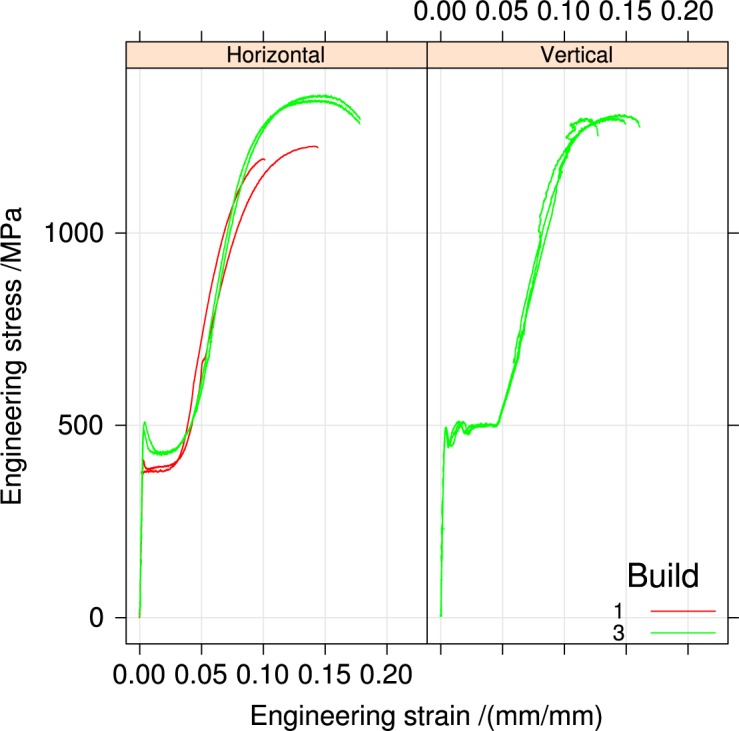
Tensile stress-strain curves for the builds, separated by orientation, for the heat-treated condition.

Figure 6 shows the orientation dependence for heat-treated specimens. Heat treatment reduces the discontinuous yielding region in the vertical specimens, but does not change the general shape.

Appendix A contains plots for all the stress-strain curves individually by build, orientation, and heat treatment for comparison.

There is evidence that plastic deformation causes a change in the phases present in the material. The undeformed material, in both the as-deposited and heat-treated conditions is non-magnetic. After deformation, the test specimens were strongly ferromagnetic.

The low-strain structure of the stress-strain curve in the discontinuous yielding region is caused by the formation and propagation of two Lüders bands, which nucleate at the ends of the gauge length and propagate until they meet in the center of the specimen. They also create the yield point behavior, Fig. 3. During the formation and propagation of the Lüders bands, the strain in the gauge section of the specimen is inhomogeneous. All the deformation is concentrated in the narrow band, and rest of the gauge section is still has not plastically deformed. Section 4.1 analyzes this behavior in greater detail.

### 3.2 Hardness

**Fig. 7:**
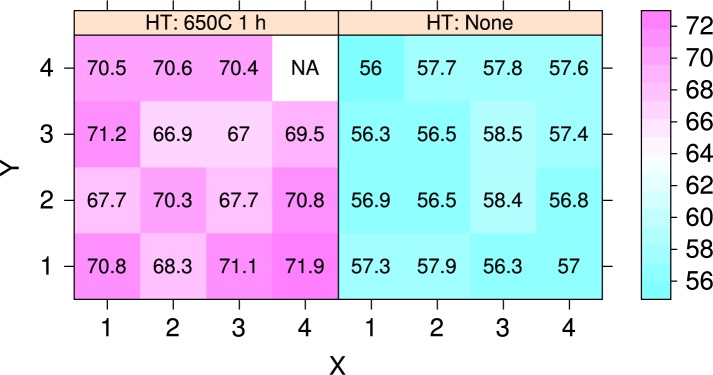
Hardness map for the sixteen hardness specimens before and after heat treatment, in units of HRA. Position (1,1) corresponds to part 1 at the front of the machine, and position (4,4) corresponds to part 16. The recoater enters from the *X* = 4 column. Color key shows hardness in units of HRA points.

**Fig. 8:**
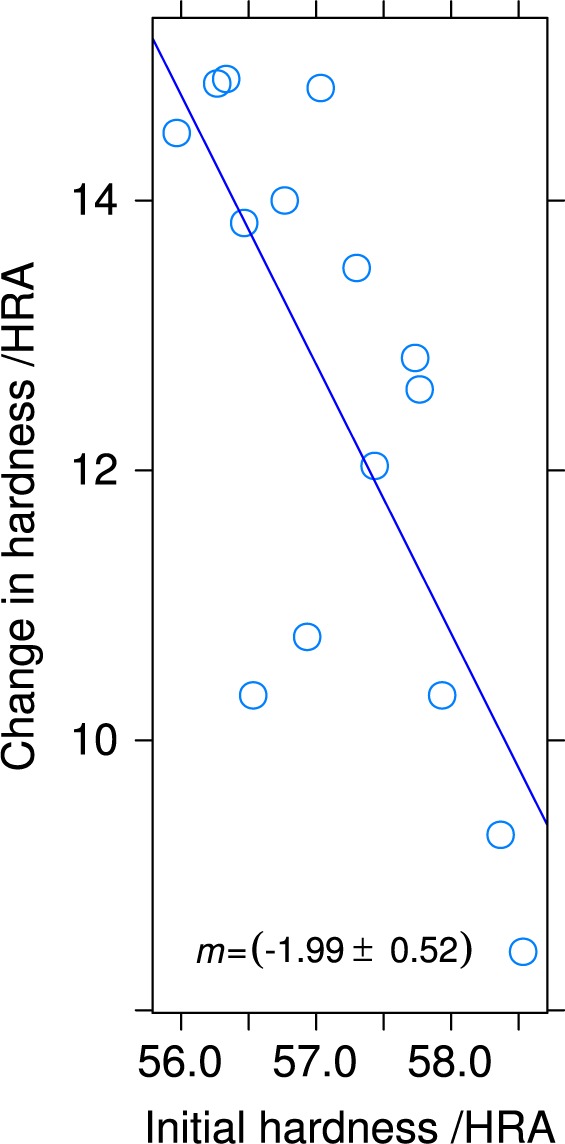
Change in hardness, in HRA points, after heat treating, as a function of initial hardness for the test specimens.

**Fig. 9:**
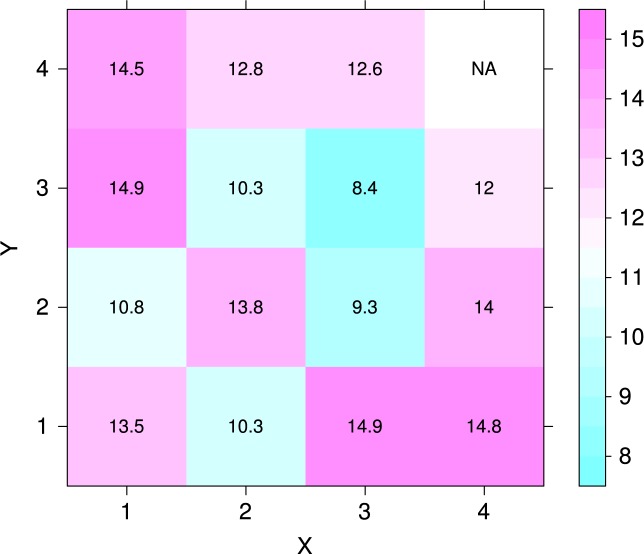
Change in hardness after heat treating as a function of position on the build plate. Color key shows the hardness change in units of HRA points.

Figure 7 plots the average hardness as a function of position on the build plate, before and after heat treatment. The hardness disks were *d* = 40 mm diameter spaced on 50 mm centers in both *X* and *Y*, and the centers of the outer rows are also 50 mm from the build-plate edge. In the plot the recoater enters from the *X* = 4 column. An analysis of variance demonstrated that there is no statistically significant difference at the 5 % confidence level between the mean hardnesses of the unheat-treated specimens. A similar analysis demonstrated that the hardnesses of specimens 4, 5, 7, 10, and 11 are significantly different from the mean hardness. Figure 8 shows that, even though the hardesses of the un-heat-treated specimens are not distinguishable, a negative correlation exists between the initial hardness and the change in hardness caused by the stress-relief heat treatment at 650 °C. Figure 9 plots the data of Fig. 8 as a function of position on the build plate. No obvious relation between hardness change and position exists. Table 2 summarizes the numerical data of Figs. 7, 8, and 9.

### 3.3 Summary Results

**Fig. 10:**
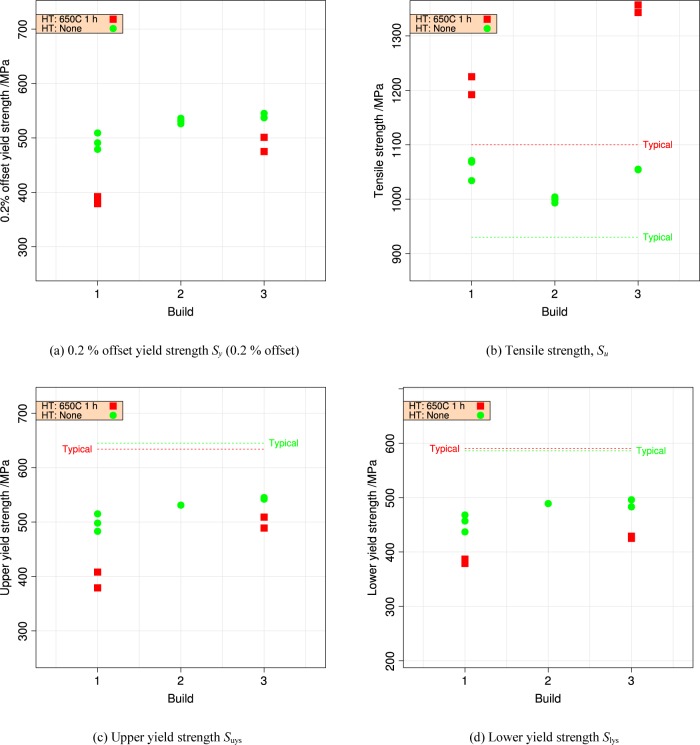
Repeatability of the yield and tensile strengths from three builds, horizontal orientation, separated by heat treatment. Horizontal lines indicate manufacturer’s [11] typical values. (a) 0.2 % offset yield strength *S_y_* (0.2 % offset) (b) tensile strength *S_u_*, (c) upper yield strength S_uys_, and (d) lower yield strength S_lys_.

**Fig. 11:**
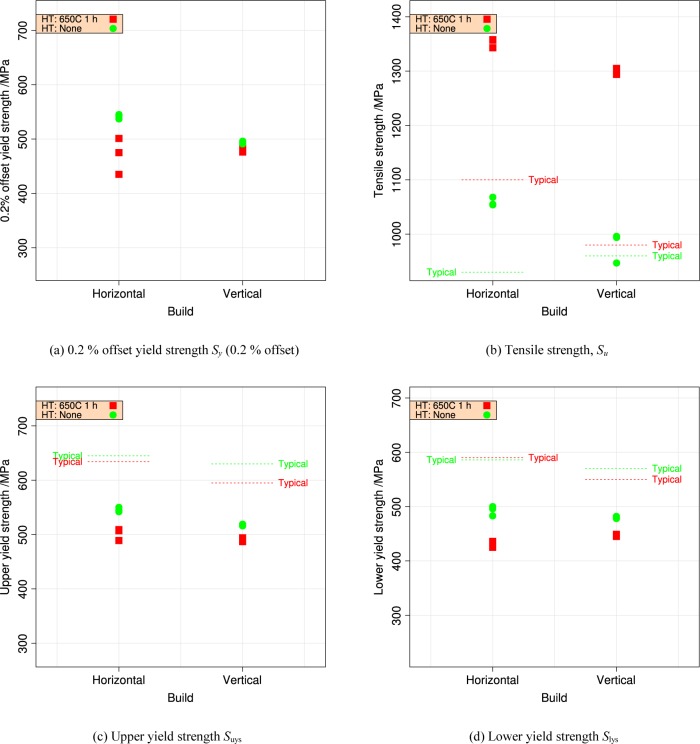
Repeatability of the yield and tensile strengths for UNS S17400 stainless steel build 2, for horizontal and vertical orientations and two heat treatments. (a) 0.2 % offset yield strength *S_y_* (0.2 % offset) (b) tensile strength *S_u_*, (c) upper yield strength S_uys_, and (d) lower yield strength S_lys_.

Table 3 summarizes the tensile data. Figures 10 and 11 plot the summary data for 0.2 % offset yield strength *S_y_* and tensile strength *S_u_*, by build and by orientation.

## 4. Analysis

### 4.1 Comparison to Accepted Values

**Fig. 12:**
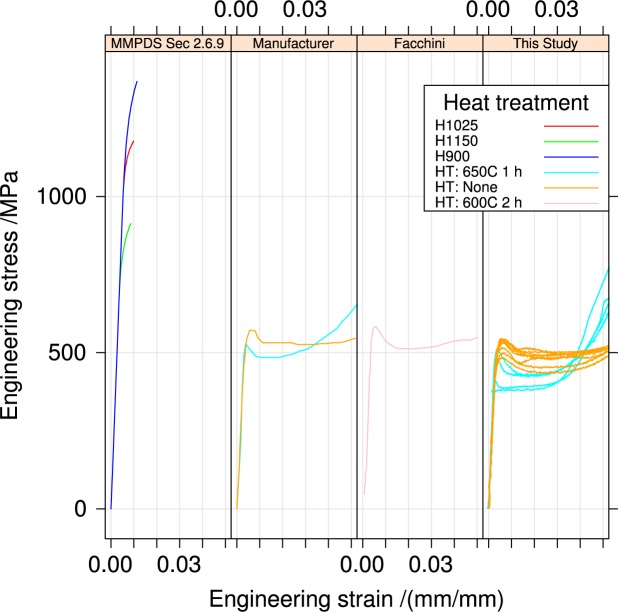
Comparison of UNS S17400 stress-strain curves to accepted behavior. Data sources: MMPDS Sec. 2.6.9 [12], Manufacturer [11], Facchini [13].

Figure 12 compares the stress-strain behavior for the UNS S17400 stainless steel in the horizontal orientation for the as-fabricated and heat-treated condition to the corresponding curves reported by the the MMPDS [12], the manufacturer [11], and one literature report from Facchini et al. [13] that used the manufacturer’s material and fabrication process. The tensile properties of the UNS S17400 stainless steel are completely different from the accepted values for 17-4PH stainless steel from the MMPDS for all heat treatments. The yield strengths of the UNS S17400 stainless steel are much lower, and the material exhibits extensive yield point elongation absent in 17-4PH stainless steel.

Figure 12 also shows that the UNS S17400 stress-strain curves from three sources are similar, but not identical. Facchini identified the origin of the difference between the “typical” values [12] for 17-4PH stainless steel and the material fabricated by selective laser melting. Wrought 17-4PH stainless steel is supplied by the mill as a fully martensitic material, which is then heat treated to form copper precipitates, which increase the strength. In contrast, Facchini et al.’s [13] analysis showed that the as-fabricated UNS S17400 stainless steel was 70 percent mass fraction metastable austenite, which transformed to the martensite phase during tensile deformation. The material of our study is similar to that of Facchini et al. [13], in that the as-fabricated material is not magnetic, and therefore not martensitic, but after deformation the specimens are strongly ferromagnetic. The deformation-induced austenite-to-martensite transformation is common in austenitic stainless steels and has been studied extensively [14,15]. Because of the different phases, it is inappropriate to refer to the UNS S17400 material as 17-4PH stainless steel, even though the powder is chemically consistent with the composition of 17-4PH SS. The UNS S17400 stainless steel fabricated by selective laser melting contains different phases–significant quantities of retained austenite as opposed to all martensite. In addition no literature sources have definitively identified Cu precipitates in the material made by selective laser melting.

### 4.2 Expected Variability of Mechanical Properties

#### 4.2.1 Strength

The powder supplier reports [16] minimum and typical properties for the upper and lower yield strengths and the tensile strength. In addition, the typical properties are reported with uncertainties of ± 50 MPa. The data sheet does not explain what this uncertainty represents, however. The specification sheet is clear that the manufacturer guarantees neither typical nor minimum properties.

Variability in mechanical properties of structural alloys comes in three forms.

1. variability in expected values for an individual alloy in a given form produced by many suppliers
2. variability introduced by differences in test protocol between the laboratories that evaluated the property
3. spatial or temporal variability within a given production run of material. In additive manufacturing, this variability might be between different areas on the build plate or between successive builds of material.

The first two examples do not represent intrinsic properties of the material. Instead, the first represents a complex combination of material processing and user expectations for materials that are often delivered to standards that specify required mechanical properties. The second represents the variability that is introduced by the attempt to measure the property itself.

**Fig. 13:**
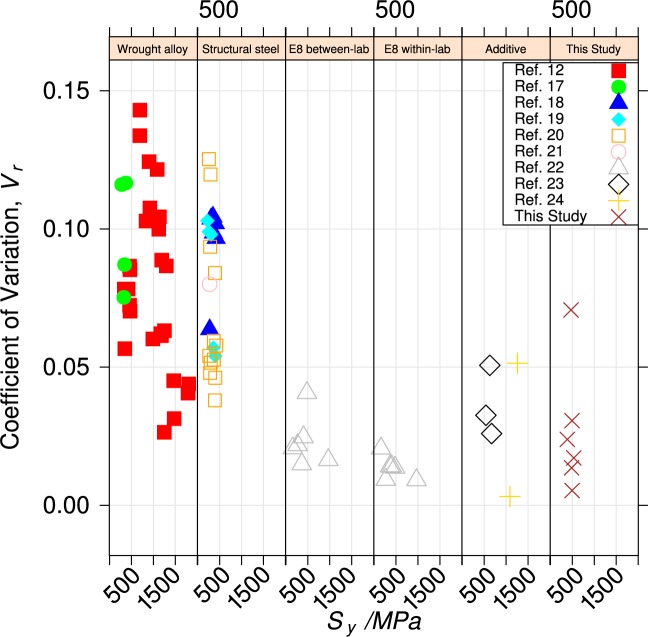
Coefficient of variation of yield strength for various alloys.

**Fig. 14:**
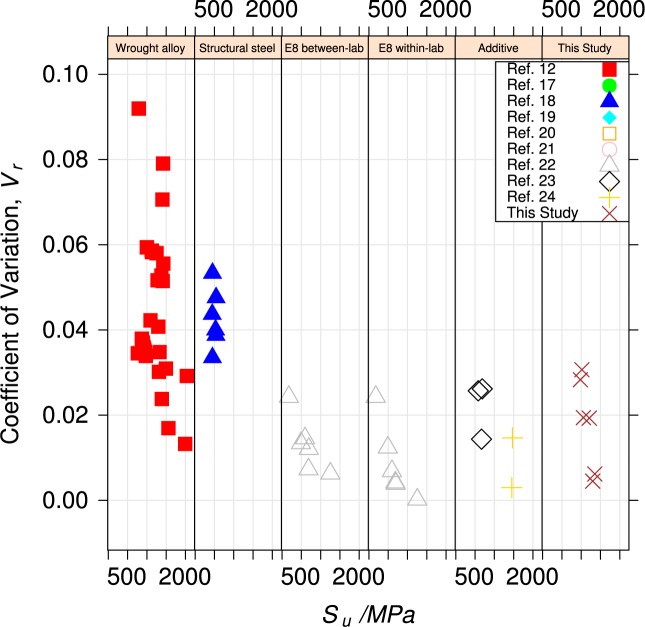
Coefficient of variation of tensile strength for various alloys.

Figures 13 and 14 compare variability for yield and tensile strengths of the UNS S17400 material of this study do that for

- aerospace and pressure-vessel ferrous wrought structural alloys, [12,17],
- structural steels for buildings [18–21],
- measurements made to evaluate the within-lab and between-lab variability of test technique [9,22], and
- two ferrous alloys made by additive manufacturing [23,24].

In the panels, the variability is expressed as the coefficient of variation, *V_r_*, defined as the standard deviation of the property normalized to its mean value.

The panel labeled “E8 within-lab” contains data [9] that was used to evaluate the variability of the ASTM E8 uniaxial tension test within a single, competent testing laboratory, using homogeneous material. Ideally, the coefficient of variability (average *V_r_* = 0.013), summarized in Table 4 represents only the contribution of the test technique itself. The variability of the material of this study and the two other ferrous alloys made by additive manufacturing are about twice as large, which indicates that some build-to-build variability exists.

The panels labeled “Wrought alloy” and “Structural steel” represent the acceptable variability for conventional mature structural engineering alloys (average *V_r_* = 0.08 also summarized in Table 4). This variability encompasses all of the within- and between-lab variability, as well as the differences in manufacturing processes.

#### 4.2.2 Orientation Effects

**Fig. 15:**
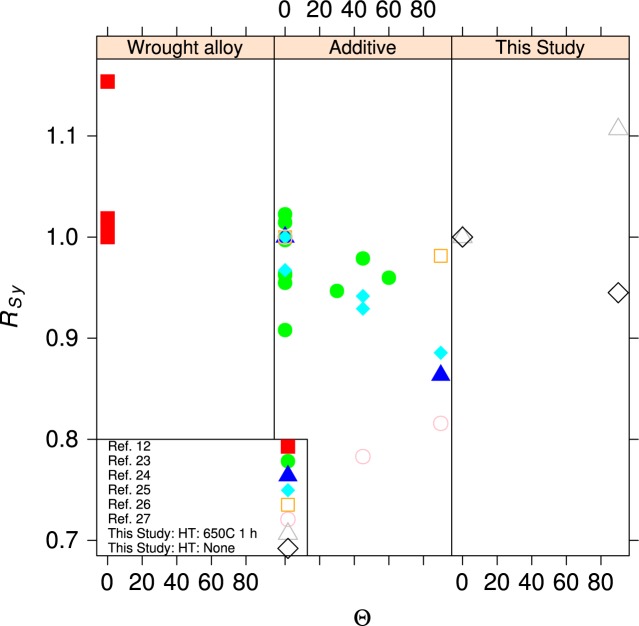
Normalized yield strength as a function angle, Θ, out of the specimen build plane.

**Fig. 16:**
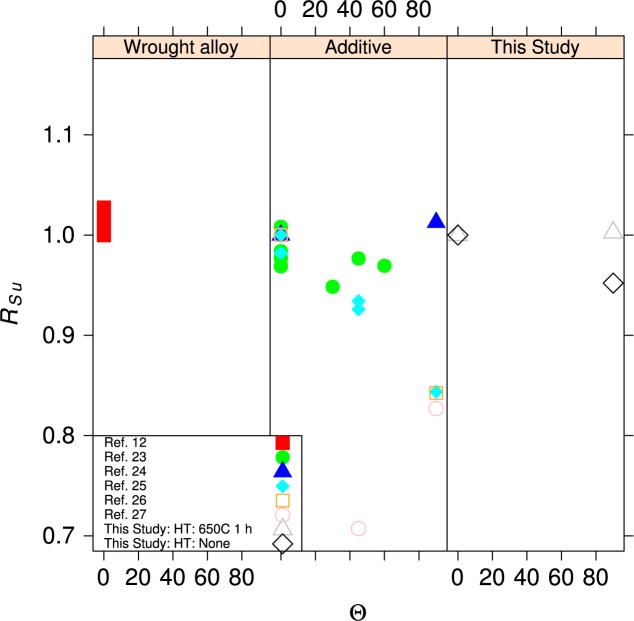
Normalized tensile strength as a function angle, Θ, out of the specimen build plane.

Metal fabrication processes rarely produce isotropic materials. Rolling a plate or bar produces a material where the strengths are reasonably equal in directions longitudinal and transverse to the rolling direction. The elongation to failure in the transverse directions can be significantly lower than in the longitudinal direction. In additive manufacturing, it is likely that the mechanical properties in the build direction will differ from those in the plane of the build, much in the same way that the directionality of rolling produces metals with anisotropic properties. Figures 15 and 16 compare the tensile and yield strengths of the material of this study to other ferrous alloys [23–27] made by additive manufacturing and selected ferrous structural alloys from the MMPDS [12]. The normalized strengths, *R*, are plotted as a function of the angle, Θ, of the specimen axis with respect to the build plane, see Fig. 2. The normalizing factor is the strength measured for an in-plane specimen (Θ = 0, Ψ = 0), so at least one data point is *R* = 1. The anisotropy of the UNS S17400 is similar to other structural alloys made by additive manufacturing, and both sets are much larger than the anisotropy expected for mature structural alloys. The data for wrought structural alloys represent longitudinal (Θ = 0, Ψ = 0) and long-transverse (Θ = 0, Ψ = 90) orientations. Short-transverse data (Θ = 90) are not available for these alloys.

### 4.3 Stress-Strain Artifacts

**Fig. 17:**
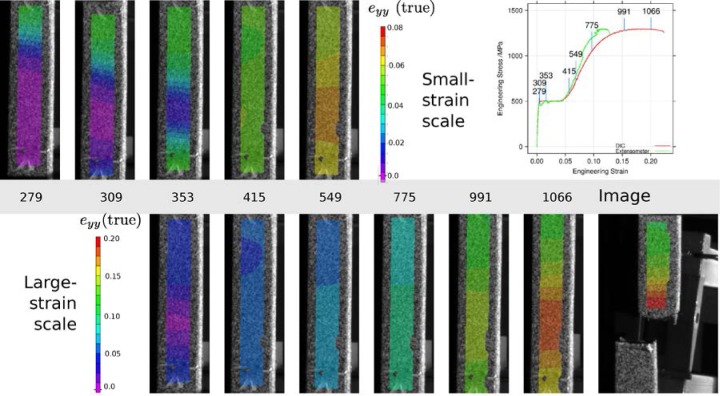
Montage of specimen images overlaid with axial strain *e_yy_*, computed from digital image correlation. The top row shows low-strain behavior, and the bottom row shows the behavior out to failure with a larger strain scale. The plot in the upper right locates each image on the stress-strain curve.

The stress-strain curves exhibit several artifacts that are known [28,29] to be created by the interaction of the discontinuous yielding or Lüders bands, the specimen geometry and surface finish, and the geometry of the extensometer. To demonstrate these artifacts, we employed digital image correlation (DIC) to map the specimen axial strain in the gauge section of a build 3 vertical heat-treated specimen. Figure 17 is a montage of images of the gauge length of the specimen at various points on the stress-strain curve. Overlaid on those images are the computed axial true strains, *e_yy_*. The top row of images shows the low-strain behavior, primarily in the region of yield point elongation. The lower row shows the behavior plotted in an expanded scale. Three images are plotted in both rows for comparison. The knife edges of the extensometer are located approximately at the ends of the region where the strains are mapped. The plot in the upper right locates the images on the stress-strain curve. It plots the engineering strain reported by the extensometer of the mechanical test system as well as the engineering strain evaluated at a point near the eventual failure location using DIC. The top row of images shows the propagation of the Lüders bands, first from the top of the specimen (Image 279) and then from the bottom of the specimen (Image 353). During the region of discontinuous yielding the center section of the specimen gauge length is strained elastically, but at each ends, the bands have produced a plastic strain *e* ≈ 0.05. The bands meet sometime before Image 415, and for a while, the strain in the gauge length is relatively uniform. Necking begins around Image 991, and is quite severe by Image 1066. The strains in the neck approach *e* = 0.2, while the strains outside the neck are only half as large. Even in Image 549, the strains in the gauge length are not uniform, and this non-uniformity manifests itself by the divergence between the extensometer-measured strain, which is essentially the average along the entire gauge length, and the DIC-measured strain, which is evaluated near the eventual fracture location.

**Fig. 18:**
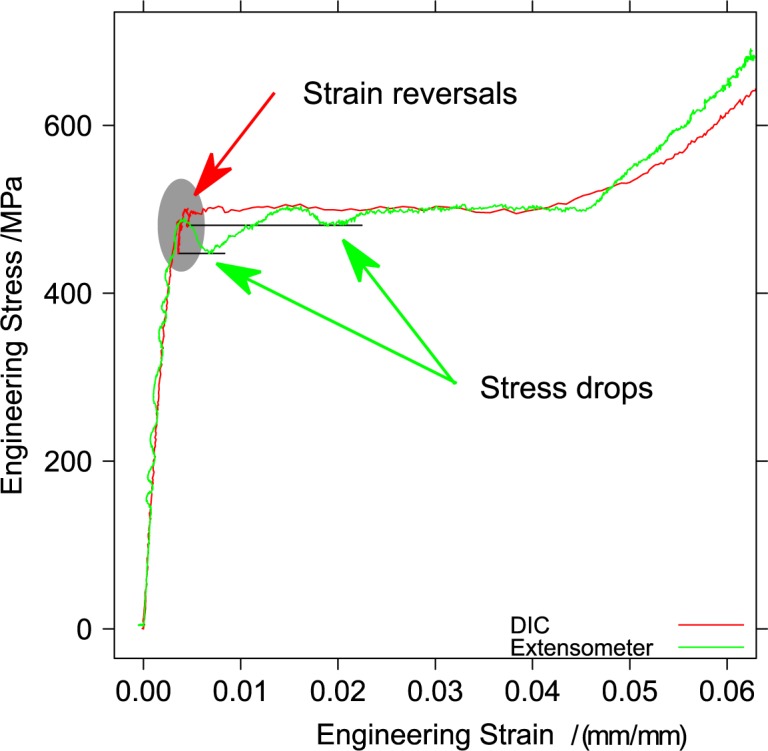
Stress-strain curve during yield point elongation measured from the extensometer and from digital image correlation of the specimen gauge section.

Figure 18 shows the region of yield-point elongation in the same test, where the axial engineering strain was evaluated by both digital image correlation and read from the test-system extensometer.

The two very repeatable dips in the engineering stress measured using the extensometer, see Fig. 6, are caused by the nucleation of the two Lüders bands. The stress-strain curve developed from the DIC data contains two force drops and strain reversals that occur just after the end of the elastic region. Because the test is conducted in position control, the instantaneous elongation produced by the nucleation of a band elastically relaxes some of the force on the specimen, and therefore a portion of the elastic strain in the region where DIC strain is calculated, which produces the strain reversals.

## 5. Summary

The results of this investigation resulted in three findings enumerated in the abstract.

1. Like wrought materials, the mechanical properties of the UNS S17400 material of this study depend on the orientation introduced by the processing: Fig. 11.
2. The recommended stress-relief heat treatment increases the tensile strength, reduces the yield strength, and decreases the extent of the discontinuous yielding: Fig. 4.
3. The mechanical properties, assessed by hardness, are very uniform across the build plate, but the stress-relief heat treatment introduced a small non-uniformity that had no correlation to position on the build plate: Sec. 3.2.

Analysis of the mechanical property behavior resulted in four conclusions:

1. The within-build and build-to-build repeatability of the tensile properties of the UNS S17400 material steel are lower than mature engineering structural alloys, but similar to other structural alloys made by additive manufacturing, Figs. 13 and 14 and Table 4.
2. The anisotropy of the mechanical properties of the UNS S17400 material of this study is larger than that of mature structural alloys, but is similar to other structural alloys made by additive manufacturing, Figs. 15 and 16.
3. The tensile mechanical properties of the UNS S17400 material fabricated by selective laser melting are very different from those of wrought, heat-treated 17-4PH stainless steel: Fig. 12.
4. The large discontinuous yielding strain in all tests resulted from the formation and propagation of Lüders bands: Fig. 17.

Finally, these findings and conclusions lead to one recommendation:

1. It is inappropriate to refer to UNS S17400 material made by additive manufacturing as 17-4PH stainless steel, because it contains different phases, which led to dramatically different mechanical properties.

## Figures and Tables

**Fig. 19:**
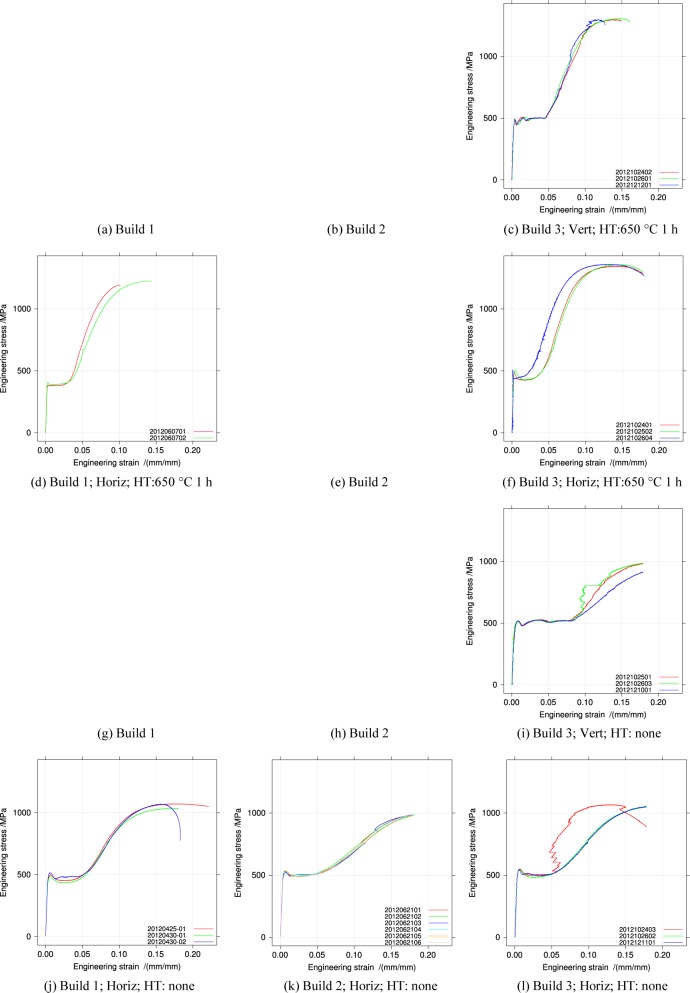
All stress-strain curves arranged by Build and heat treatment. No data was taken in the missing entries.

**Table 1:** Number of specimens in each build round, with orientations and heat treatments.

Build	As deposited			Heat-treated		
	Horizontal		Vertical	Horizontal		Vertical
	Hp	Hv	V	Hp	Hv	V
1	x	3	x	2	x	x
2	6	x	x	x	x	x
3	3	x	3	3	x	3

**Table 2:** Results of all hardness tests.

Position (*X*,*Y*)	Part	Heat Treatment	
		None HRA	650 °C 1 h HRA
(1, 1)	1	53.9, 59, 59	70, 71.3, 71.1
(2, 1)	2	56, 59, 58.8	67.1, 67.5, 70.2
(3, 1)	3	55.1, 56.9, 56.8	73, 70.5, 69.9
(4, 1)	4	57.8, 55.4, 57.9	73.5, 71.5, 70.6
(1, 2)	5	56.9, 54.9, 59	66.3, 68.6, 68.2
(2, 2)	6	55.5, 58.4, 55.5	71.6, 68.5, 70.8
(3, 2)	7	61.6, 55, 58.5	66.4, 69.4, 67.2
(4, 2)	8	55.7, 56.6, 58	69, 69.4, 73.9
(1, 3)	9	56.1, 57.8, 55.1	71.5, 71.9, 70.3
(2, 3)	10	56.9, 54.9, 57.8	67.6, 66.2, 66.8
(3, 3)	11	60.5, 58.1, 57	67.5, 67.7, 65.7
(4, 3)	12	56.9, 58.5, 56.9	67.9, 68.3, 72.2
(1, 4)	13	57.5, 55.1, 55.3	70.4, 69.6, 71.4
(2, 4)	14	59.5, 56.8, 56.9	69.6, 69.9, 72.2
(3, 4)	15	59.1, 57, 57.2	68.3, 70.6, 72.2
(4, 4)	16	57.5, 58.2, 57.1	

**Table 3:** Summary data for the tensile tests: *S_y_* = 0.2% offset yield strength; strength units are MPa and strain rate units are mm/mm/s.

Build	*S_y_*	*S*_uys_	*S*_lys_	*S_u_*	*de*/*dt*	Heat Treatment	Orientation
1	491	498	457	1071	6.7E-05	HT: None	Horizontal
1	479	483	437	1034	6.6E-05	HT: None	Horizontal
1	509	515	468	1068	6.6E-05	HT: None	Horizontal
1	379	379	379	1192	6.5E-05	HT: 650C 1 h	Horizontal
1	392	408	387	1225	6.5E-05	HT: 650C 1 h	Horizontal
2	531	531	489	999	8.3E-04	HT: None	Horizontal
2	526			993	8.3E-04	HT: None	Horizontal
2	514			986	8.3E-04	HT: None	Horizontal
2	536			1004	8.3E-04	HT: None	Horizontal
2	532			1001	8.3E-04	HT: None	Horizontal
2	531			1006	8.3E-04	HT: None	Horizontal
3	475	489	425	1343	9.1E-05	HT: 650C 1 h	Horizontal
3	542	550	500	1068	9.1E-05	HT: None	Horizontal
3	501	509	429	1357	9.1E-05	HT: 650C 1 h	Horizontal
3	537	542	483	1054	9.1E-05	HT: None	Horizontal
3	435	507	436	1358	9.1E-05	HT: 650C 1 h	Horizontal
3	545	545	496	1055	9.1E-05	HT: None	Horizontal
3	476	494	445	1296	9.1E-05	HT: 650C 1 h	Vertical
3	496	519	482	996	9.1E-05	HT: None	Vertical
3	489	493	447	1305	9.1E-05	HT: 650C 1 h	Vertical
3	495	516	480	994	9.1E-05	HT: None	Vertical
3	484	487	449	1294	9.1E-05	HT: 650C 1 h	Vertical
3	491	516	478	947	9.1E-05	HT: None	Vertical

**Table 4:** Coefficient of variation for various alloy classes.

Class	*V_r_* (*S_y_* (0.2 % offset))	*V_r_* (*S_u_*)
Wrought alloy	0.080	0.044
Structural steel	0.079	0.043
E8 between-lab	0.023	0.013
E8 within-lab	0.013	0.009
Additive	0.033	0.017
This Study	0.027	0.018

## 6. Appendix A. Stress-Strain Curves

Figure 19 plots all a matrix of all the stress-strain curves differentiated by build, orientation, and heat treatment. Because some builds did not produce both orientations or both heat-treated specimens, some of the entries in the matrix are blank. Some specimens produced erratic extensometer readings (build 3 no heat-treatment). The erratic measurements are caused by the extensometer slipping on the specimen. In most cases the extensometer was removed when it reached the limit of its extension and before failure.
